# Tooth Loss-Associated Mechanisms That Negatively Affect Cognitive Function: A Systematic Review of Animal Experiments Based on Occlusal Support Loss and Cognitive Impairment

**DOI:** 10.3389/fnins.2022.811335

**Published:** 2022-02-10

**Authors:** Xiaoyu Wang, Jiangqi Hu, Qingsong Jiang

**Affiliations:** Department of Prosthodontics, Beijing Stomatology Hospital, School of Stomatology, Capital Medical University, Beijing, China

**Keywords:** tooth loss, cognitive dysfunction, Alzheimer's disease, vascular dementia, neurodegenerative diseases, oxidative stress, mitochondrial autophagy, nerve damage

## Abstract

**Background:**

There is a dose-response relationship between tooth loss and cognitive impairment, while tooth loss can be an independent risk factor for Alzheimer's disease (AD) and vascular dementia (VaD). Tooth loss can also accelerate nerve damage and neurodegeneration. However, the associated mechanisms remain poorly understood.

**Objective:**

To conduct a systematic review of animal experiments on cognitive decline caused by the loss of occlusal support performed over the past 10 years and summarize the possible underlying mechanisms.

**Methods:**

“Tooth Loss,” “Edentulous,” “Tooth Extraction and Memory Loss,” “Cognition Impairment,” and “Dementia” were used as keywords to search PubMed, Embase, SCI, ScienceDirect, and OpenGrey. A total of 1,317 related articles from 2010 to 2021 were retrieved, 26 of which were included in the review after screening according to predetermined inclusion and exclusion criteria. Comprehensiveness was evaluated using ARRIVE guidelines and the risk of bias was assessed using SYCLE'S risk of bias tool.

**Results:**

The putative mechanisms underlying the cognitive impairment resulting from the loss of occlusal support are as follows: (1) The mechanical pathway, whereby tooth loss leads to masticatory motor system functional disorders. Masticatory organ activity and cerebral blood flow decrease. With reduced afferent stimulation of peripheral receptors (such as in the periodontal membrane) the strength of the connections between neural pathways is decreased, and the corresponding brain regions degenerate; (2) the aggravation pathway, in which tooth loss aggravates existing neurodegenerative changes. Tooth loss can accelerates nerve damage through apoptosis and mitochondrial autophagy, increases amyloid deposition in the brain; and (3) the long-term inflammatory stress pathway, which involves metabolic disorders, microbial-gut-brain axis, the activation of microglia and astrocytes, and inflammatory cascade effect in central nervous system.

**Conclusion:**

The loss of occlusal support may lead to cognitive dysfunction through the reduction of chewing-related stimuli, aggravation of nerve damage, and long-term inflammatory stress.

## Introduction

There are many risk factors for cognitive disorders such as Alzheimer's disease (AD) and vascular dementia (VaD). Included psychosocial factors such as depressive symptoms, limited interests, major traumatic life events, lack of education, poor economic status, smoking, and alcohol abuse. And biological risk factors, such as advanced age, sex, apolipoprotein E4 (APOE4) allele, hypertension, hyperlipidemia, diabetes, and heart disease. In decades, an increasing number of studies have shown that a bidirectional relationship exists between the loss of occlusal support and cognitive dysfunction. Patients with cognitive impairment tend to neglect their oral hygiene, and are more likely to develop periodontitis (Chu et al., 2015; Campos et al., 2017; Ma et al., 2021) and to experience tooth loss at an earlier age. However, the loss of occlusal support can significantly increase the risk of dementia (Del Brutto et al., 2014; Dintica et al., 2018), accelerate neurodegeneration, and directly lead to cognitive impairment. Although this connection has been confirmed at the epidemiological level, the associated mechanisms remain unclear. In this review, we systematically discuss and summarize three putative underlying pathways identified through the screening of relevant animal studies undertaken over the last 10 years.

## Methods

### Search Strategy

Older patients with degenerative dementia (such as AD, Parkinson's disease, and Lewy body dementia) account for the largest proportion of people with cognitive impairment, followed by patients with vascular dementia, and then other types of dementia. Tooth loss can lead to cognitive dysfunction, while a thorough understanding of the associated pathogenesis can benefit the treatment and/or prevention of this condition. Animal models are conducive to determining the mechanisms that might link the loss of occlusal support and cognitive impairment at different ages.

Food quantity and texture can be strictly controlled in animal experiments. Because powder feeding is also considered to reduce chewing-related stimuli and mimic tooth loss, it was also included as an intervention. Memory loss is often used as a predictor of mild cognitive impairment and dementia. Thus, in addition to “cognitive impairment,” keywords related to memory loss were also used as search strings.

**Figure 1:**
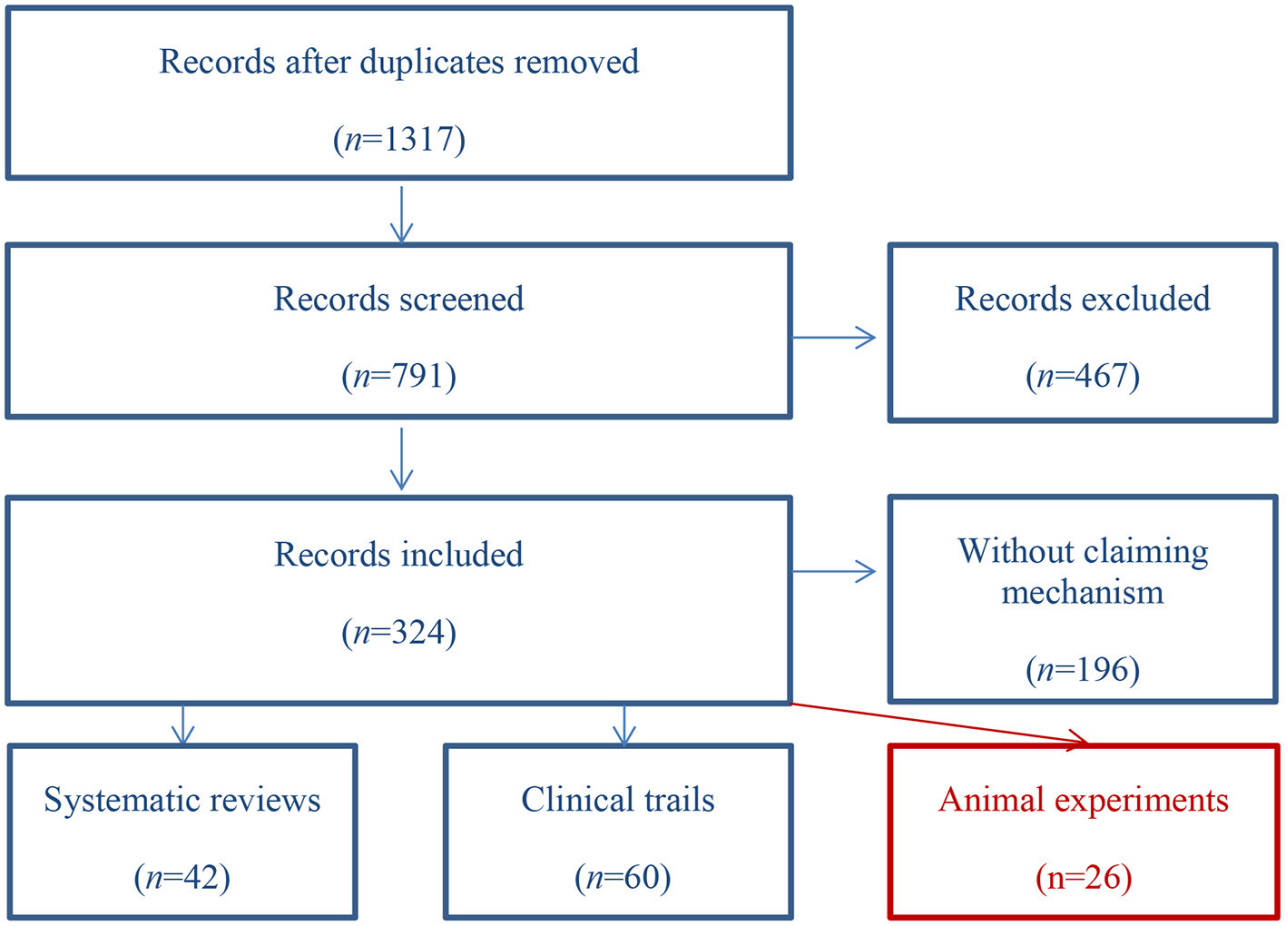
Scanning path.

A literature search was performed in PubMed, Embase, SCI, ScienceDirect, and OpenGrey for related articles published between 2010 and 2021 (the search string is shown in Table 1). Article selection was carried out by two researchers (the scanning path is shown in Figure 1). Any disagreement was resolved by a third researcher from the same group. A total of 1,317 records were identified, 526 of which were excluded due to duplication. After screening the titles and abstracts, 467 articles were excluded as they did not meet the inclusion criteria. Of the remaining 324, 128 (40%) met the inclusion criteria, most of which were clinical trials and reviews. Finally, 26 articles involving animal experiments were included for analysis.

**Table 1:** Search string.

**Database**	**Search string**	**Results**
PubMed	((tooth loss) OR (edentulous) OR ((tooth extraction) OR (tooth extract) OR (molar extract) OR (incisor extraction) OR (incisor extract) OR (molar extraction))) AND ((cognition impairment) OR (dementia) OR ((memory loss) OR (hypomnesia)) OR (Alzheimer)). Filters: published in the last 10 years	299
Embase	(('tooth'/exp OR tooth) AND ('loss'/exp OR loss) OR (edentulous) OR (('tooth'/exp OR tooth) AND ('extraction'/exp OR extraction)) OR (('tooth'/exp OR tooth) AND ('extract'/exp OR extract)) OR (('molar'/exp OR molar) AND ('extract'/exp OR extract)) OR (('incisor'/exp OR incisor) AND ('extraction'/exp OR extraction)) OR (('incisor'/exp OR incisor) AND ('extract'/exp OR extract)) OR (('molar'/exp OR molar) AND ('extraction'/exp OR extraction))) AND (('cognition'/exp OR cognition) AND ('impairment'/exp OR impairment) OR 'dementia'/exp OR dementia OR (('memory'/exp OR memory) AND ('loss'/exp OR loss)) OR hypomnesia OR Alzheimer)	659
SCI	TS=((“tooth loss” OR “edentulous” OR “tooth extraction” OR “molar extraction”) AND (“cognition impairment” OR “dementia” OR “memory loss” OR “hypomnesia” OR “Alzheimer”)) Databases = WOS, BCI, CSCD, DIIDW, KJD, MEDLINE, RSCI, SCIELO. Timespan = 2010–2021. Search language = English	314
ScienceDirect	((tooth loss) OR (edentulous) OR (tooth extraction)) AND ((cognition impairment) OR (dementia) OR ((memory loss) OR (hypomnesia)) OR (Alzheimer))	44
OpenGrey	((tooth loss) OR (edentulous) OR ((tooth extraction) OR (tooth extract) OR (molar extract) OR (incisor extraction) OR (incisor extract) OR (molar extraction))) AND ((cognition impairment) OR (dementia) OR ((memory loss) OR (hypomnesia)) OR (Alzheimer))	1

### Inclusion Criteria

(i) Type of experiment: *in vivo* animal experiment.
(ii) Subjects: experimental animals.
(iii) Publication time: January 2010–September 2021.
(iv) Control group: present.
(v) Experimental intervention: tooth extraction/soft food provision.
(vi) Results: behavioral and central nervous system-related changes.
(vii) Following damage to the inferior alveolar nerve, the neural pathways of the corresponding teeth were damaged, which was consistent with the hypothesis that “tooth loss leads to reduced afferent nerve stimulation.” Thus, damage to the inferior alveolar nerve was included in the study.

### Exclusion Criteria

(i) Articles reporting the grinding of short teeth were excluded as the influence of residual periodontal membrane in mastication cannot be ruled out (Pei et al., 2018).
(ii) Articles reporting experiments using implants/prosthodontics for missing teeth were also excluded.
(iii) The association between periodontal inflammation and cognition has been widely confirmed (Ishida et al., 2017; Iwasaki et al., 2019; Xue et al., 2020); however, the underlying mechanism remains unclear. The experiment of periodontal disease vs. cognitive impairment is excluded.

### Comprehensiveness of Scientific Reporting and Risk of Bias

Selection bias can be analyzed from three aspects: sequence generation, baseline characteristics, and allocation hiding. Only one article reported dividing the animals into groups according to whether there was cognitive decline based on the results of a passive avoidance test (Oue et al., 2013); none of the other articles assessed the baseline levels of individual cognition. Therefore, it was assessed as “uncertain.” Implementation bias was judged as “uncertain” because none of the experiments clarified whether the animal breeder also performed the experiment. Measurement bias was not clarified. The effect of other biases was low in the 26 included studies.

## Results

A total of 26 articles met the inclusion criteria and could be divided into the following three categories according to the associated mechanism: (1) Reduction in mechanical masticatory stimulation and weakening of afferent nerve stimulation; (2) aggravate degeneration; and (3) chronic inflammatory stress. Compared with clinical trials, animal experiments are easier to “randomize” and perform “blind.” However, for subsequent clinical studies, the risk of bias in animal experiments needs to be correctly assessed. Table 2 shows the internal authenticity assessment of the included reports based on the SYRCLE risk of bias assessment tool for animal experiments. A detailed evaluation indicated that the comprehensive bias risk of the included studies was low and that the comprehensiveness and authenticity were reliable. The included literature is summarized in Table 3 (mechanical pathway), Table 4 (aggravation pathway), and Table 5 (inflammatory stress pathway).

**Table 2:** Risk of bias assessment.

**Bias**	**Risk assessment**
Selection bias	Uncertain
Implementation bias	Uncertain
Check out the bias	Low
Lost to bias	Low
Reporting bias	Uncertain
Other bias	Low

**Table 3:** The mechanical pathway.

**Species/amount**	**Grouping (*N*)**	**Age**	**Behavioral experiment**	**Results**	**Other experiments**			**Results**
					**Sample**	**Method**	**Marker analyzed**	
KM mice	Occlusal contact control group (20) Right lateral mastication group (20) Left side masticatory group (20)	Tooth extraction at 6 weeks; behavioral test 4/8 weeks after tooth extraction	Morris water maze test Passive avoidance experiment	There was no difference in the water maze test at 4 weeks, and the unilateral mastication group performed poorly in the active avoidance test. There were significant differences between the two groups at 8 weeks (the left tooth extraction group performed the worst).	Hippocampus	HPLC RT-PCR WB	(1) 5-HT (2) 5-HT1A/BDNF/TrkB (3) 5-HT1A/5-HT2A	(1) The level of 5-HT was lower in the unilateral chewing group (the reduction in 5-HT1A levels in the right tooth extraction group was specific). (2, 3) Gene expression decreased at 4 weeks/8 weeks in the right tooth extraction group and at 8 weeks in the left tooth extraction group
Jiang et al. (2021)							
C57BL/6J mice	I/S group [maxillary molars intact + hard food (12)] E/S group [maxillary molars extracted + hard food, (12)] I/P group [maxillary molars intact + meal (12)] E/P group [maxillary molars extracted + powder food (12)]	Tooth extraction at 28 weeks; behavioral test 4/16 weeks after tooth extraction	Passive avoidance experiment	No memory impairment at 4 weeks The experimental group showed memory decline at 16 weeks	Hippocampus hypothalamus	RT-PCR IHC	BDNF/TrkB expression levels Number of pyramidal cells in the CA1/CA3 regions of the hippocampus	At 4 weeks, there were significant differences in BDNF expression levels between the extraction and non-extraction groups and between the soft and hard food groups in the hippocampus. No differences were found in the hypothalamus. At 16 weeks, the expression of BDNF was decreased in the hippocampi of mice in the tooth extraction + soft food group. No differences were found in the hypothalamus No differences in the numbers of pyramidal cells were observed among the groups at 4 weeks. At 16 weeks, the numbers of pyramidal cells in the CA1/CA3 regions were significantly decreased in the molar extraction groups; however, no significant differences were detected between mice fed a soft or a hard diet
Takeda et al. (2016)							
C57BL/6J mice	Normal diet group Powder food group	Feed of different hardness at 3 weeks	Passive avoidance test Object location memory task open field test Rotating test	The group fed hard food performed better in the passive avoidance test	Femur/jawbone, chewing muscles, hippocampus	RT-PCR IHC	BDNF/Ntrk2 expression levels Number of neuronal precursor cells Number of c-Fos-positive cells	BDNF/TrkB expression decreased in the soft diet group The density of precursor neurons in the dentate gyrus decreased in the soft diet group Synaptic formation in the hippocampus was reduced in the soft diet group
Fukushima-Nakayama et al. (2017)							
Wistar rats	Edentulous group [extraction of all maxillary molars (16)] Control group [anesthesia + sham operation (16)]	Tooth extraction at 3 months Morris water maze test 12 weeks after surgery	Morris water maze test	The edentulous group performed poorly	*In vivo*/*in vitro* Hippocampus (CA1)	ASL–MRI HPLC RT-PCR IHC	Cerebral blood flow Glutamate levels, Bax/Bcl-2/caspase-3 expression Number of pyramidal cells in the hippocampal CA1 region	Twelve weeks after tooth extraction, hippocampal blood flow in the edentulous group was significantly lower than that in the control group The hippocampal glutamate content in the edentulous group was significantly higher than that of the control group; Bax/Bcl-2/caspase-3 levels were significantly higher in the edentulous group than in the control group There were significantly fewer pyramidal cells in the hippocampal CA1 region of rats in the edentulous group relative to the control group
Luo et al. (2019)							
Sprague–Dawley rats	Experimental group [extraction of the left upper and lower molars (30)] Control group [anesthesia and gingival division (30)]	Tooth extraction at 3 months; examination 1 week after surgery; behavioral tests 8 weeks after tooth extraction	Gambling task open field test	There was no effect on the movement ability of the rats after tooth extraction. Rats in the experimental group received less food. In the experimental group, the proportion of poor decision makers increased; (evaluation of decisions depended on the distribution of correct decisions throughout the test)	*In vivo*/*in vitro* Brain	Multi-electrode recording IHC	Local electric field potentials (LFPs); anterior cingulate cortex (ACC) and basolateral amygdala (BLA) signals ACC and BLA related electrode trajectory	In the experimental group, theta wave activity decreased in the ACC, but increased in the BLA. Functional connectivity between the ACC and the BLA decreased and there was only loose interaction.
Xu et al. (2015)							
SAMP8 mice	Extraction group (extraction of maxillary molars) Control group (no teeth extracted)	Tooth extraction at 8 weeks; behavioral tests at 12/24 weeks	Morris water maze test	Cognitive impairment occurred earlier in the experimental group. The experimental group showed a preference for the position of objects	None	None	None	None
Kawahata et al. (2014)							
Wistar rats	Tooth extraction group [all maxillary molars removed (10)] Restoration group [denture restoration after extraction (10)] Control group [blank (10)]	Tooth extraction at 7 weeks; restoration at 11 weeks; experiment at 50 weeks	Eight-arm radial maze test	Error crossing times: extraction group > restoration group > control group	Brain left hippocampus (CA1, CA3)	IHC	Pyramidal cell density in the hippocampus	The density of pyramidal cells in the CA1 area was in the order of extraction group < restoration group < control group. The density of pyramidal cells in the CA3 area was lower in the extraction and restoration groups than in the control group
Kurozumi et al. (2019)							
Wistar rat	Experimental group [botulinum toxin type A (BTXA) injection (10)] Control group [injection of normal saline (10)]	Injection at 4 weeks; euthanasia after 28 days	None	None	Hippocampus	IHC	CREB/p-CREB Neuronal density	CREB/p-CREB expression decreased in the experimental group Neuronal density in the CA1/CA3 regions and dentate gyrus (DG) decreased in the experimental group
Tsai et al. (2018)							
SAMP8 mice	Sham operation group (7) Extraction group [extraction of all maxillary molars (13)]	Tooth extraction at 22 months; experiment 3 months after surgery	Open field test	No difference	Cerebral cortex hippocampus	WB	BDNF/TrkB	BDNF expression decreased significantly in the extraction group no difference was found for TrkB expression
Jiang et al. (2011)							
C57BL/6J mice	Each part of the experiment was divided into separate groups	Unclear	None	None	Kidney, masticatory muscle, trigeminal nerve, hippocampus Mouse myoblasts (C2C12)	WB, RT-PCR Electrical stimulation of the masticatory muscle Tracing of near-infrared (NIR) dye-labeled exosomes	NEP WB IHC	Masticatory muscle expressed high levels of NEP protein and mRNA. NEP produced by masticatory muscle was absorbed by trigeminal ganglion and transported to the hippocampus both *in vivo* and *in vitro*
Kobayashi et al. (2019)							
BALB/c mice	Hard feed group (25 g hard food/week) Soft feed group (25 g powder food/week)	Feeding began from 24~44 weeks and lasted for 1 month	None	None	Olfactory bulb brain mitral cells	Patch clamp recording	Action potentials	The recorded action potentials were generated by GABA synapses. The frequency and amplitude of mitral cell action potentials in the soft diet group were lower than that in the hard diet group
Noguchi et al. (2017)							
SAMP8 mice	Age: 4 months (40), randomized into middle-aged experimental groups 1 and 2 and middle-aged control groups 1 and 2 Age: 7 months (20), randomized into an elderly experimental group and an elderly control group. In the experimental groups, the inferior alveolar nerve was exposed and ligated. In the control groups, it was only exposed	Middle-aged experimental and control groups 1: at 8 months Middle-aged experimental and control groups 2: at 11 months Elderly experimental and control groups: at 11 months	Step-down test Y-maze test	In the learning stage, the performance of the elderly experimental group was significantly worse compared with the other groups. There was no difference between the test groups The learning rate of the elderly experimental group was lower than that of the elderly control group. There was no difference among the middle-aged groups.	Brain	IHC LM	CA1/CA3 pyramidal cell count ChAT-immunoreactive neurons AChE-positive nerve fibers	There were significantly fewer pyramidal cells in the hippocampi of mice in the elderly experimental group relative to those of mice in the elderly control group, and the arrangement was disordered. There were significantly fewer ChAT-immunoreactive neurons in the septal nuclei of mice in the elderly experimental group. There were also significantly fewer AChE-positive nerve fibers in the CA1 region and dentate gyrus of the elderly experimental group than in those of the elderly control group; no difference was detected among the other groups.
He et al. (2014)							
SAMP8 mice	Experimental group (tooth extraction) Control group (blank)	Tooth extraction at 1 month; experiment at 8 months	Morris water maze test	Spatial memory and learning ability were impaired in the experimental group	Plasma hippocampus	ELISA IHC LM	New cells in the dentate gyrus Synapses	The number of surviving and proliferating cells was decreased in the dentate gyrus. Synaptophysin expression was inhibited in the hippocampus
Kubo et al. (2017)							
CD1 mice (3–4 months old)	Experimental group (extraction of left upper and lower molars) Control group	Experiment 4 weeks after tooth extraction	None	None	Brain	IHC	New neurons labeled with dual anti-corticosteroid/anti-neuronal nuclear antigen antibodies	Compared with the control group, dicorticosterone-/nGA-positive cells in the dentate gyrus of the experimental group were fewer and scattered. The synapses were short and discontinuous
Su et al. (2014)							

**Table 4:** The aggravation pathway.

**Species/ amount**	**Grouping (N)**	**Age**	**Behavioral experiments**	**Results**	**Other experiments**			**Results**
					**Sample**	**Methods**	**Marker analyzed**	
C57BL/6J mice 3 × Tg-AD mice	Extraction group (bilateral extraction of maxillary molars) Control group (sham operation)	Tooth extraction at 4 months; brain separated at 2/4/7 months; behavioral tests at 4/5/6/7/8 months	Barnes maze test	The experimental group performed significantly worse	Trigeminal nucleus Mesencephalon hippocampus	IHC retrograde transport marker	Number of neurons Aβ42/CD86	The number of neurons in the trigeminal nucleus, locus coeruleus, and hippocampus decreased. The number of CD86-immunoreactive microglia increased after Aβ42 aggregation
Goto et al. (2020)								
Female transgenic AD mice (J20)	Experimental group [bilateral extraction of maxillary molars (10)] Control group [blank (7)]	Tooth extraction at 6 months; behavioral test 4/6 months after surgery	Passive avoidance test (according to the experimental results, the mice were divided into a memory maintenance group and an impaired memory group)	Memory was impaired in the experimental group	Serum left brain hippocampus	ELISA IHC LM	Serum corticosterone, Aβ42/Aβ40 levels Aβ deposition Hippocampal pyramidal cell density	There was no significant difference in serum corticosterone levels or Aβ42/Aβ40 expression in the brain. There was no significant difference in Aβ deposition in the brain. The pyramidal cells in the CA1/CA3 regions of the hippocampus were significantly reduced in the experimental group, but there was no difference in the range. Pyramidal cell reduction was associated with poorer performance in the avoidance test
Oue et al. (2013)								
Female Tg2576 mice	Experimental group [bilateral maxillary molars extracted (13)] Control group [anesthesia only (10)]	Tooth extraction at 14 months; experiment 4 months after surgery	Passive avoidance test	No significant difference	Brain	ELISA IHC LM	Aβ40/Aβ42 levels Aβ deposition Hippocampal pyramidal cell density	Aβ40 expression was not detected and Aβ42 levels differed significantly different between groups. Aβ deposits were detected in both groups, but there was no significant difference. There was no significant difference in the number of hippocampal CA1 and CA3 pyramidal cells between the groups.
Oue et al. (2016)								
APP knock-in mice	Experimental group [maxillary molar extraction (8)] Control group [sham operation (8)]	Experiment 4 months after surgery	Morris water maze test	The experimental group had longer escape latency and shorter target quadrant cruise time	Plasma hippocampus	ELISA WB IHC	Plasma corticosterone/Aβ42/ Aβ40 levels Aβ deposition	There was no significant difference in plasma corticosterone content, Aβ42/Aβ40 levels, or Aβ deposition. Some cognitive impairment due to loss of occlusal support was found but did not seem to be associated with Aβ deposits
Murakami et al. (2021)								
ICR mice	Normal-fed control at 3 weeks group (12) Extraction at 3 weeks group (11) Zinc deficiency (ZD) at 3 weeks group (12) Extraction at 3 weeks + zinc deficiency (EZD) group (11) Normal-fed control at 4 weeks group (12)	Tooth extraction at 3 weeks; zinc-deficient diet until 12 weeks (zinc: 48.9 μg/g); normal diet resumed at 12 weeks; the behavioral test was followed by 5 weeks of recovery	Modified water maze test (8, 13, 22 weeks of age; the experiment was conducted directly in 4-week-old mice)	A zinc-deficient diet had a significant effect on the escape latency of rats at the ages of 13 and 22 weeks. There was no significant difference in the number of platform search failures	Hippocampus	IHC	Astrocytes	ZD/EZD group was significantly higher than Ext/C group in CA1 region. There is no difference in the remaining area
Kida et al. (2015)								

**Table 5:** The inflammatory stress pathway.

**Species/ amount**	**Grouping (*N*)**	**Age**	**Behavioral experiment**	**Results**	**Other experiments**			**Results**
					**Sample**	**Method**	**Marker analyzed**	
Wistar rats	Chronic cerebral ischemia group 2-VO (16) Cerebral ischemia sham operation group 2-VO (16) Occlusal support loss group M (16) Occlusal support loss sham operation group MS (16) Control group C (16)	Sham/tooth extraction at 3 months; experiment after 8 weeks	Morris water maze test	The spatial learning and memory abilities of the 2-VO and M groups were impaired, but there was no difference between the two groups	Hippocampus	Griess assay WB IHC	NO iNOS/eNOS iNOS/eNOS	NO release was higher in the 2-VO and M groups; more iNOS (+) cells were found in the 2-VO and M groups; more eNOS (+) cells were found in the 2-VO and M groups. Immunohistochemical staining results were consistent with those mentioned above.
Pang et al. (2020)							
KM mice	Maxillary extraction group (E1, 15) Maxillary sham operation group (S1, 15) Mandibular extraction group (E2, 15) Mandibular sham operation group (S2, 15)	10–11 months	Morris water maze test	In the experimental group, the exploration trajectory was disordered and the number of times passing the target quadrant was significantly reduced.	Prefrontal cortex, hippocampus	Griess assay (8 weeks after extraction), IHC WB	Body weight NO/iNOS	There was no difference in body weight after 1 week In the experimental group, NO/iNOS release was higher in the hippocampus than in the prefrontal cortex
Pang et al. (2015)							
Wistar rats	Extraction group [bilateral maxillary molar extraction, (10)] Post-extraction restoration group (10) Control group (sham operation after general anesthesia)	Tooth extraction at 7 weeks; restoration at 41 weeks; behavioral test at 50 weeks	Eight-arm radial maze test	The error rate of the D3 pre-extraction group was significantly higher than that of the control group. The error rate of the D8 anterior denture restoration group was significantly lower than that of the extraction group. After D13, there was no difference between groups.	Serum, hippocampus (dentate gyrus + CA1–3)	ELISA IHC	Serum corticosterone levels Cell density in the hippocampus	There was no difference in serum corticosterone levels. The cell density of each area of the hippocampus was in the order of control group > repair group > tooth extraction group
Sakamoto et al. (2014)							
SAMP1 mice	Upper and lower molar extraction group (10) Maxillary molar extraction group (10) Mandibular molar extraction group (10) Soft food control group (10) Hard food control group (10)	Anesthesia and tooth extraction at 5 weeks	The amount of activity during the alternation of day and night	There was no difference in the initial stage, but there was an intergroup difference after 12 weeks	None			
SAMP8 mice	Edentulous group [all maxillary molars removed (7)] Control group [anesthesia only (7)]	Tooth extraction at 1 month; recording 8 months later	None	None	Serum brain	ELISA LM TEM	Body weight/daily food intake Serum corticosterone levels Cell morphology in hippocampal regions Organelle morphology	There was no difference in body weight or food intake during the experiment. Serum corticosterone content in the edentulous group was significantly higher than that in the control group. There was no significant difference in pyramidal cell morphology in the hippocampus. Mitochondrial damage and the density and quantity of lipofuscin in the cytoplasm both increased in the edentulous group
Katano et al. (2020)							
APPNL-G-F mice	Experimental group (all maxillary molars were extracted; ♂4 ♀4) Control group (anesthesia only; ♂4 ♀4)	Tooth extraction at 2 months; powder feeding 2 months after surgery; behavioral tests after 2 months	Novel object recognition test Passive avoidance test	In the experimental group, learning disorders were detected at the age of 7 months; both short-term and long-term memory were impaired	Brain, cerebral cortex, hippocampus	IHC ELISA WB RT-PCR	Aβ40/Aβ42/nerve cells/glial cells; Aβ40/Aβ42; BCA/TNF-α/IL-6/IL-1β/IL-10/TGFβ; p-CREB/p-ERK	There was no difference in insoluble Aβ40/Aβ42 levels in the hippocampus and cerebral cortex. In the experimental group, the numbers of c-Fos- positive neurons in the hippocampus and cerebral cortex decreased significantly; p-CREB levels decreased, but p-ERK levels increased; glial cells were activated in the hippocampus and cerebral cortex; and the number of pyramidal cells decreased
Taslima et al. (2021)							
SAMP8 mice	Standard tooth extraction group Tooth extraction with enhancement group Control group Control enhancement group	Tooth extraction at 8 months; breeding in a standard or enhanced environment 3 weeks after surgery	Morris water maze test	The experimental group had impaired spatial memory and learning ability	Hippocampus	EM	Morphological characteristics of myelin sheaths and synapses in the hippocampus	The myelin sheaths of CA1 neurons became thinner and the density of postsynaptic neurons decreased
Kubo et al. (2021)							

### Animal Models

Rodents were used in all 26 studies assessed. Twenty-one studies entailed the extraction of molars, most of which involved bilateral maxillary molar extraction, and the remainder unilateral molar extraction. Notably, no anterior teeth were extracted in any of the included studies because the effect on animal nutrition intake is believed to be too severe. The age of the animals ranged from 3 weeks to 12 months. In two studies, food hardness was altered to adjust the masticatory intensity to simulate masticatory disorders following the loss of occlusal support. He et al. (2014) exposed and truncated the inferior alveolar nerve of middle-aged and elderly mice and assessed the distribution of hippocampal cells and the behavior of the animals after 4 months. The results showed that injury to the inferior alveolar nerve can damage the hippocampus and affect cognition.

Six of the studies did not perform behavioral experiments. The Morris water maze test was the most frequently used behavioral test, followed by the passive avoidance, novel object recognition, and open field tests.

### The Mechanical Pathway

Mastication is an autonomous rhythmic movement generated by a central pattern generator (CPG) located in the pons and medulla. Receptors in the cerebral cortex and peripheral receptors together help regulate the formation of a unique chewing pattern. Anatomically, the upper craniofacial and maxillofacial areas are closely related. The loss of occlusal support can change the blood supply in the brain and affect cognitive ability through vascular-related factors. Hasegawa et al. (2007) showed that mastication can increase cerebrovascular blood flow, while Miyake et al. (2012) reported that chewing exercise can help prevent Alzheimer's disease by maintaining a good blood supply to the brain. The above relationship was further confirmed in animal experiments. Using arterial spin labeling–magnetic resonance imaging (ASL-MRI), Luo et al. (2019) found that blood flow in cognition-related brain areas was significantly decreased 12 weeks after tooth extraction in rats. Concomitantly, the glutamate content and the expression levels of *Bax/Bcl-2, caspase-3*, and other apoptosis-related genes in the hippocampal CA1 region were increased, whereas the number of pyramidal cells was decreased. Meanwhile, in the behavioral experiment, rats that underwent tooth extraction showed memory impairment.

Studies have shown that removing molars at a young age leads to a reduction in the number of new cells in the mouse hippocampus and cognitive decline in young mice. Jiang et al. (2011) suggested that afferent stimulation might be weakened after tooth loss, thereby resulting in the down regulation of the BDNF/TrkB/CREB signaling pathway and synaptic plasticity in the hippocampal CA1 region. The loss of occlusal support affects the entire cognition-related network, not only one brain region. Xu et al. (2015) compared the electrophysiological characteristics of the anterior cingulate cortex (ACC) and basolateral amygdala (BLA) between controls and rats subjected to molar extraction after electrical stimulation and found that theta frequency, the characteristic signal of cognition, was asynchronous between the ACC and BLA after tooth extraction. This indicated a weakening of the connection between the ACC and BLA and that tooth extraction exerted a negative effect on the strength of neural networks.

Masticatory organs produce nutritional factors that are retrogradely transported through nerves. Kobayashi et al. (2019) performed electrical stimulation of masticatory muscles *in vitro* and *in vivo* to simulate muscle contraction and then tracked labeled neprilysin (NEP) produced by masticatory muscles. The results showed that NEP was transmitted from masticatory muscles to the hippocampus *via* the trigeminal nucleus in C57BL/6J mice. The authors suggested that this enzyme, which facilitates the clearance of the amyloid-beta (Aβ) peptide, can be retrogradely transported to the brain during masticatory muscle contraction.

### The Aggravation Pathway

Tooth loss has been identified as a risk factor for AD. Tooth loss may accelerate neurodegeneration by increasing Aβ deposition in the brain. The earlier tooth loss occurs, the more pronounced the neurological effects seem to be. Goto et al. (2020) undertook an immunohistochemical analysis and a Barnes Maze spatial learning/memory assessment at different time points after the bilateral extraction of the maxillary molars in triple transgenic AD (3 × TG-AD) mice and found that cytotoxic Aβ42 was released in the trigeminal nucleus and locus coeruleus after 4 months. This resulted in a significant reduction in the number of neurons in the hippocampal CA1/CA3 regions that receive projections from the locus coeruleus. Similarly, Oue et al. (2013) analyzed amyloid deposition and neuron numbers in the hippocampi of 6-month-old J20 mice 4 months after tooth extraction and found no difference in Aβ, Aβ40, and Aβ42 contents between the experimental and control groups. However, differences in behavioral performance and hippocampal nerve number were detected.

### The Inflammatory Stress Pathway

Tooth loss induces the activation of inflammatory cells. Taslima et al. (2021) reported that, after tooth extraction in mice with amyloid precursor protein (*App*) gene knock-in (a mouse model of AD), the numbers of microglia and astrocytes in the hippocampus increased, and a large number of inflammatory cytokines (such as tumor necrosis factor alpha [TNF-α], interleukin-1 [IL-1], and IL-6) were produced, thereby promoting an inflammatory state in neurons in the hippocampus and prefrontal cortex. Early tooth loss can also lead to malnutrition and chronic stress. Kubo et al. (2021) extracted maxillary molars from SAMP8 mice soon after tooth eruption (at 1 month of age) and found that the plasma corticosterone content was significantly higher in the experimental group than in the control group, whereas synaptophysin expression was inhibited. Most researchers in the 26 studies measured plasma corticosterone levels and correlated plasma corticosterone content with chronic stress, thereby quantifying the negative effects of tooth loss on animals, at least partially.

## Discussion

Based on a total of 26 animal-based studies from 2010 to 2021, the objective of this review was to summarize the putative mechanisms underlying the cognitive dysfunction caused by tooth loss. Clinically, this condition primarily affects patients with AD, and treating such patients is always complicated. The existing therapeutic regimens can only reduce memory loss and delay disease deterioration, they cannot cure the disease. Therefore, intervention in the early stages of memory loss is expected to yield the most effective results. A small number of epidemiological studies found no association after confounding factors were removed between tooth loss and cognitive impairment (Naorungroj et al., 2015; Stewart et al., 2015), not animal-based studies. A larger number of epidemiological studies have shown that tooth loss can be a risk factor for Alzheimer's disease and other cognitive disorders (Cerutti-Kopplin et al., 2016; Tsai et al., 2020; Wang and Ge, 2020), and there is a dose-response relationship between the number of missing teeth and cognitive impairment (Chen et al., 2018).

If the link between tooth loss and cognitive impairment is confirmed, it will provide a novel means of targeting the prevention of cognitive impairment. Many clinical studies have shown that chewing exercise can be considered as a preventive method for cognitive impairment (Ono et al., 2010; Tada and Miura, 2017; Chuhuaicura et al., 2019). Corresponding results appeared after the use of implants or dentures to repair the missing teeth, patients' cognitive level can be improved (Ki et al., 2019; Tan et al., 2020). Avivi-Arber et al. (2015) have proved that the neural plasticity of the facial primary motor cortex (FACE-M1) and the adjacent primary somatosensory cortex (FACE-S1) of rats was enhanced after the implantation. More animal-based experiments on observation of cognitive performance after restoration are needed.

Here, we conducted a comprehensive evaluation of the literature according to the ARRIVE checklist and SYRCLE risk of bias assessment tool. The network connecting tooth loss and cognitive impairment is complex, and the analysis of a single pathway often leads to vague and confusing results. Therefore, most of the studies evaluated in this study included multiple mechanisms of action simultaneously.

Most of the articles reported that changes in cognition-related brain regions after tooth loss resulted in impaired masticatory movement and reduced oral stimulation. Cerebral blood flow is stable in general. However, studies have confirmed that mastication has positive effect on cerebral blood flow (Sesay et al., 2000; Luo et al., 2019), increasing blood flow in the motor area, somatosensory area, thalamus and cerebellum, and the number of pyramidal cells in the hippocampus (Momose et al., 1997; Stratulat et al., 2014). And it leads to changes in cerebral metabolism through glial cells. For example, astrocytes wrap around vascular walls and nerves and are activated after cerebral ischemia (Koizumi et al., 2018). It plays a neuroprotective role by ingesting glutamate, releasing glutamine to reduce oxidative stress damage, repairing blood brain barrier (BBB) (Neuhaus et al., 2014), and so on. Activation of the glial cell network accompanied by release of a large number of cytokines and neurotoxic reactive oxygen species (ROS), which can lead to cognitive decline through PI3K/AKT/mTOR and many other pathways (Yang et al., 2021).

The teeth and periodontal membrane are supplied by various branches of the trigeminal nerve. After unilateral damage on different branches of trigeminal nerve in rats, the amount of ganglion neuron on both side was counted. Eight percent associated with teeth (Gregg and Dixon, 1973). Reduction of dental pulp- and periodontal membrane-derived neural signals may have an important negative impact on cognition-related brain regions (Taylor et al., 2009; Osborne et al., 2018). The regulation of mastication and sensation involves multiple brain regions, including the brain stem, prefrontal cortex, thalamus, and limbic system, as well as communication among neural networks related to cognition (Lin et al., 2017). After extraction of incisors and fangs in cat, afferent nerve fibers in the trigeminal nucleus reduced (Linden and Scott, 1989), the number of neurons and the postsynaptic density decreased in the hippocampal CA1 region (Katano et al., 2020). Brain-derived neurotrophic factor (BDNF) and its related pathways may play a certain role in this process (Huang et al., 2021). BDNF is one of the most widely studied neurotrophic factors, produced by nervous system and dominant target organs [such as muscle, dental pulp, and salivary gland (Saruta et al., 2020)]. It is mainly expressed in the hippocampus, cerebral cortex and other cognition-related brain regions, assisting development and repairing of the nervous system. Tyrosine Kinase receptor B (TrkB) is a specific receptor for BDNF. BDNF/TrkB participate in autophagy or neuroplasticity through PI3K/Akt, MAPK/Ras and other pathways (Bramham and Messaoudi, 2005), and eventually have influence on learning and memory. The essential role of BDNF in exercise-memory link may inspire us on the mechanism research between mastication and cognition (Loprinzi and Frith, 2019).

Mitochondrial damage and oxidative stress are the two most reliable hypotheses about aging. Occlusal support loss induces cognitive dysfunction by aggravating degeneration in brain. Early tooth loss leads to mitochondrial damage in hippocampus (Katano et al., 2020). Tooth loss causes excessive releasing ROS (Zhao et al., 2021) and reactive nitrogen free radicals (RNS), let oxidation exceeds the removal of oxides, resulting in tissue damage. Tooth loss can also activate the immune-related glial cells that produce a large number of bioactive factors, such as TNF-α, IL-1, IL-3, IL-6, colony-stimulating factor (CSF), and Interferon-γ (IFN-γ) in brain. Tooth loss leads to neuroplasticity changes and apoptosis (Del et al., 2013; Sofroniew, 2013). Clinical (Okamoto et al., 2010; Goto and Leung, 2019) and animal studies (Oue et al., 2013; Goto et al., 2020) related to Alzheimer's disease, have confirmed the aggravating effect of missing teeth on brain degeneration; however, when it comes to animal modeling, the analyzed genes are mostly related to familial Alzheimer's disease, which accounts for <5% of the total number of these patients (Cannon-Albright et al., 2019), highlighting the need for the development of a more appropriate model to investigate this condition.

Tooth extraction and local anesthesia are strong traumatic stimuli (Gasparini et al., 2002), and the long-term loss of teeth puts the body in a state of chronic stress (Singhrao et al., 2014). Chronic stress is accompanied by changes in glucose, fat and protein metabolism. With further research on cognitive disorders in the last decades, metabolic disorders are gradually regarded as the pathogenic mechanism behind dementia represented by AD (Manyevitch et al., 2018; Ryu et al., 2019). Fasting blood glucose rises sharply under chronic stress, even meets the diagnostic criteria of stress diabetes (Halim, 2019). Elderly patients with diabetes risk from dementia (Ahtiluoto et al., 2010; Biessels and Despa, 2018). This may be related to insulin resistance or dysglycemia in brain. Dysglycemia of brain observed with fluorodeoxyglucose positron emission tomography (FDG-PET) can be one of the valuable indicators long before AD. Besides, enhanced lipolysis brings more free fatty acids and ketone bodies into blood, Ketone bodies can go through the BBB and provide energy to brain. Though several human studies have shown that ketogenic diet had a cognitive protective effect (Neth et al., 2020; Davis and Fournakis, 2021), the relationship between cerebral ketone body and cognitive disorders remains to be studied. From another point of view, enhanced mastication can inhibit the hypothalamic-pituitary-adrenal (HPA) axis (Azuma et al., 2017) and attenuate stress-induced increases in the plasma concentrations of glucocorticoid, catechol, and nitric oxide (NO) (Sunariani et al., 2019). They can cross the BBB and lead to depression, anxiety, and cognitive disorders.

Chronic stress is closely related to inflammation. After teeth extraction, chronic stress activates glial cells and release excess ROS, causing an inflammatory cascade effect in the central nervous system (Taslima et al., 2021). Chronic inflammation will also interact with chronic stress and oxidative stress, thereby aggravating nerve damage. In clinical practice, tooth loss is mostly caused by periodontitis. Large number of periodontal pathogens (such as Klebsiella) can be ingested and disturb the intestinal flora (Olsen and Yamazaki, 2019; Xue et al., 2020). Through the microbial-gut-brain axis, short-chain fatty acids (SCFAs) produced by intestinal flora participate in recruiting neutrophils, dendritic cells (DCs), macrophages, T cells and other immune cells, and arise systemic immune responses (Silva et al., 2020; Mirzaei et al., 2021). SCFAs activate glial cells network and cause central nervous inflammation. The relationship between tooth loss caused by severe periodontitis and cognitive impairment along the microbial-gut-brain axis remains to be further studied.

**Figure 2:**
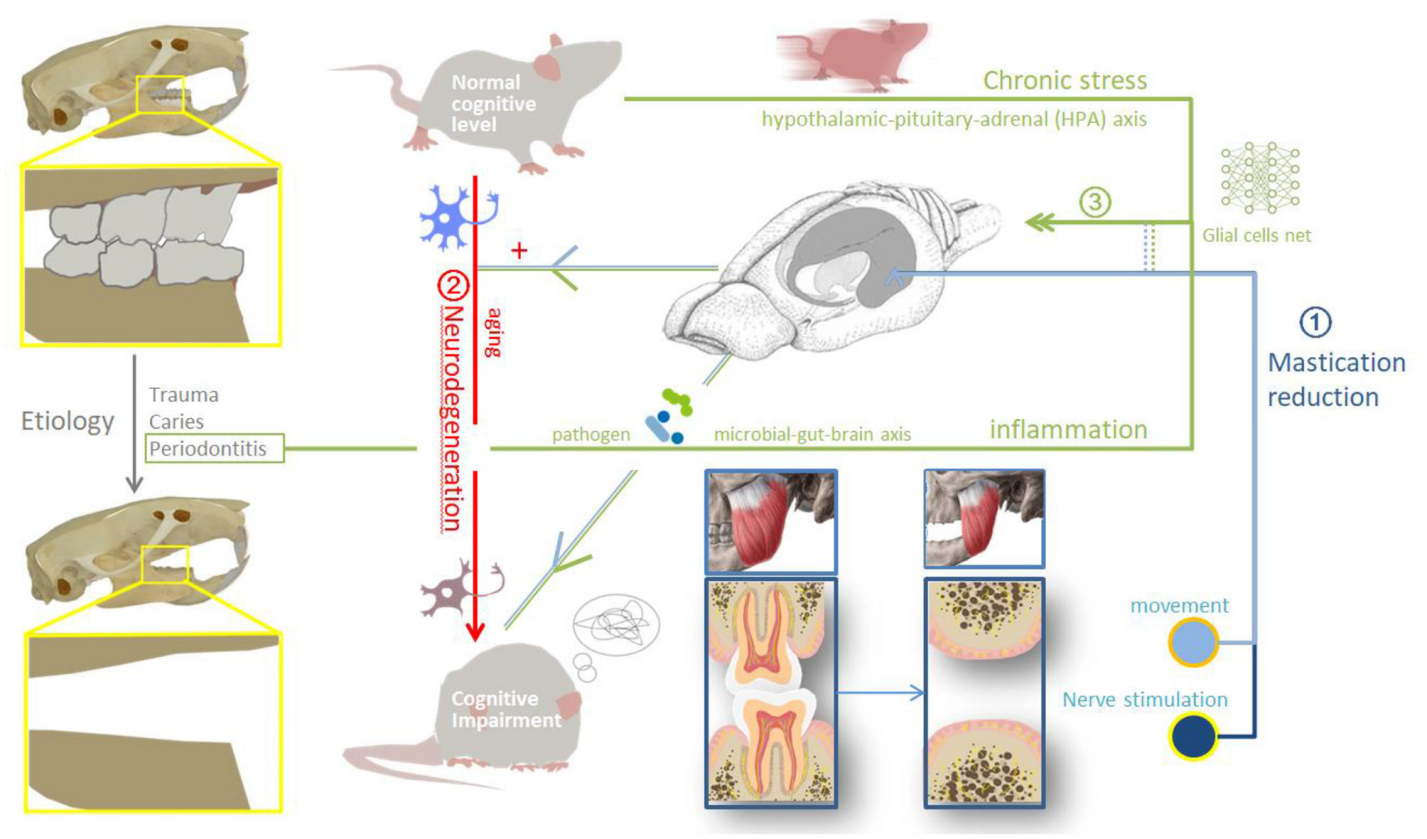
Possible mechanisms.

In conclusion, the loss of occlusal support can lead to cognitive impairment through three mechanisms: impaired masticatory movement, the aggravation of neurodegenerative changes, and long-term inflammatory stress (The possible mechanisms are shown in Figure 2). The pathogenesis, even the most commonly studied cognitive disorders such as AD, remains unclear at present. In the future, mechanism of tooth loss leading to cognitive impairment needs to catch up with the evolution of brain science. Specific cytological changes after tooth loss or screening of sensitive indicators before cognitive impairment require cohort study. Further studies are needed on the cognitive changes before and after implantation or restoration of losing teeth *in vivo*.

## Data Availability Statement

The datasets presented in this study can be found in online repositories. The names of the repository/repositories and accession number(s) can be found in the article/supplementary material.

## Author Contributions

XW: literature searching—screening and manuscript writing. JH: design searching strategy and literature screening. QJ: evolution of searching aims. All authors have given approval to the final version of the manuscript.

## Funding

This work was supported by the National Natural Science Foundation of China (Grant numbers 81771094 and 82170980).

## Conflict of Interest

The authors declare that the research was conducted in the absence of any commercial or financial relationships that could be construed as a potential conflict of interest.

## Publisher's Note

All claims expressed in this article are solely those of the authors and do not necessarily represent those of their affiliated organizations, or those of the publisher, the editors and the reviewers. Any product that may be evaluated in this article, or claim that may be made by its manufacturer, is not guaranteed or endorsed by the publisher.
